# Assessment of the Effect of Phosphorus in the Structure of Epoxy Resin Synthesized from Natural Phenol–Eugenol on Thermal Resistance

**DOI:** 10.3390/ijms27010112

**Published:** 2025-12-22

**Authors:** Danuta Matykiewicz, Beata Dudziec, Adam Piasecki

**Affiliations:** 1 Faculty of Mechanical Engineering, Poznan University of Technology, Piotrowo 3, 61-138 Poznan, Poland; 2 Faculty of Chemistry and Center for Advanced Technologies, Adam Mickiewicz University in Poznan, Uniwersytetu Poznanskiego 10, 61-614 Poznan, Poland; beata.dudziec@gmail.com; 3 Faculty of Materials Engineering and Technical Physics, Poznan University of Technology, al. Jana Pawla II 24, 61-138 Poznan, Poland; adam.piasecki@put.poznan.pl

**Keywords:** eugenol, resin, flame, degradation, epoxy

## Abstract

This work aimed to investigate the thermal properties of phosphorus-modified epoxy resin obtained from eugenol derivatives and cured with different amines: aliphatic—triethylenetetramine (TETA); aromatic—diaminodiphenylmethane (DDM); and cycloaliphatic—isophoronediamine (IDA). The thermal stability was investigated through both thermogravimetric analysis (TGA) coupled to a Fourier transform infrared spectrometer (TGA/FTIR) and pyrolysis–combustion flow calorimetry (PCFC). The structures of the cured castings and the char residues were assessed by scanning electron microscopy (SEM). Eugenol-based resin during thermal degradation is covered with a significant amount of char residue and is characterized by a reduced value of heat release rate (HRR) and heat release capacity (HRC) compared with the resin based on petrochemicals.

## 1. Introduction

To meet the stringent requirements of engineering materials, epoxy resins are modified with various flame-retardant additives, reactive or non-reactive, dispersed in the matrix [1,2]. The combustion process is a very complex chemical reaction involving heat and mass exchange, for which the necessary factors are combustible substances, oxygen, and heat [3]. Modification of epoxy resins with melamine [4], montmorillonite [5], boron [6], phosphorus [7], silicon [8], or layered double hydroxides [9] improves thermal stability and flame retardancy. However, the modification process requires the selection of specific methods for mixing and processing the epoxy material. Due to the low toxicity of the gases evolved during combustion, organophosphorus-based compounds are a favorable solution in the field of fire resistance of epoxy resins. The organophosphorus compound is first converted to phosphoric acid by combustion and thermal degradation. A further heat treatment then produces nonvolatile polyphosphoric acid, which ultimately reacts with the degrading polymer through esterification and dehydration to help form char residues [10].

Therefore, a promising solution is the use of phosphorus-modified plant raw materials [11] to produce materials with increased fire resistance [12] and flame retardants [13]. To improve the fire resistance of polymers, chemical modification, polymerization, and incorporation of inert material or additive fillers into the chemical structure are used [1,14]. The synthesis and application of a bio-based flame retardant based on eugenol (DPSi-ED) and dihydro-9-oxa-10-phosphaphenanthrene-10-oxide (DOPO) was described by Li et al. [15]. This flame retardant was incorporated into the epoxy/4,4′-diaminodiphenylmethane (DDM) system. At the same phosphorus content (0.5 wt%), epoxy/DPSi-ED thermosets indicated enhanced flame resistance. Furthermore, epoxy/DPSi-ED7 reached a limiting oxygen index (LOI) of 35.3% and a V-0 rating in the UL-94 tests, and compared to epoxy/ED7, its peak heat release rate (pHRR) and total smoke production (TSP) were reduced by 31.5% and 10.9%, respectively. It was proved that the abundant benzene ring in DPSi-ED contributed to the char layer, which was stabilized by the synergistic effect of phosphorus and silicon elements. Furthermore, Gan et al. described a bio-derived eugenol-containing phosphorus silicone hybrid molecule (EGDS-DOPO) that provides excellent flame retardancy and enhanced mechanical performance to epoxy/amine materials [2]. The addition of only 5 wt% of EGDS-DOPO to the epoxy matrix allowed it to achieve an LOI of 34.9% and a UL 94 V-0 rating. Cone calorimetry analysis showed a reduction in pHRR, the total heat release (THR), and TSP by 62.2%, 34.0%, and 47.5%, respectively, when the EGDS-DOPO concentration was 20% by weight, compared to the unmodified epoxy resin. Ecochard et al. prepared a series of eugenol-based epoxy monomers by nucleophilic substitution of phosphorus-containing POCl_3_, POCl_2_Ph, or POCl_2_(OPh) and epoxidation of its allylic group [16,17]. The inclusion of phosphorus as a linker between phenols conferred flame-retardant properties over epoxy resin cured with meta-xylylenediamine, without the use of flame retardants. The introduction of phosphorus influenced a decrease in pHRR and THR but also led to a decrease in degradation temperatures. These phenomena are common when phosphorus is added, especially phosphonate and phosphate groups, which may act as char promoters. It was also observed that the decomposition of phosphorus-modified materials was more complex. Lei et al. obtained eugenol–phosphazene epoxy resin (EHPE) by nucleophilic substitution of eugenol and hexachlorocyclotriphosphazene, followed by epoxidation of the allyl group of eugenol [18]. Polyetheramine-cured EHPE showed an LOI value of 31%, reached the UL-94V-0 rating, and had a T_g_ value of 122 °C. The char layer indicated intumescent properties, and the PHRR value of EHEP decreased significantly by nearly 66% compared to the unmodified epoxy resin. Yu et al. applied the eugenol–siloxane–phosphorus molecule (EGN-Si-P) to epoxy resin (DGEBA) cured with DDM and obtained an LOI value of 29.7% and an UL-94 V-0 rating [19]. Chen et al. prepared a flame-resistant bio-based epoxy resin monomer described as BEEP using eugenol and phenylphosphonic dichloride [20]. Bio-based phosphorus-containing flame-retardant epoxy resins were obtained by combining BEEP at various concentrations with commercial epoxy resin cured with 4-aminophenyl disulfide (APDS). Improved properties such as an LOI of 27.4% and a UL-94 V-0 grade were achieved when the ratio of epoxy resin to BEEP was 7:3. However, Cao et al. developed a biomass-based phosphorus cocuring agent (VFD) derived from vanillin, furfurylamine, and DOPO. Epoxy resin with 7.5 wt% (only 0.52 wt% phosphorus was loaded) was used as an additive to achieve a V-0 rating with an LOI of 34.5% and favorable mechanical properties [21]. Also, Liu et al. synthesized a highly efficient flame retardant for epoxy from lignin-derived vanillin and guaiacol (DGEBDB) containing DOPO units. DGEBDB was mixed with the commercial epoxy resin (DGEBA), cured with DDM, and acted as a reactive fire-resistant [22]. The UL-94 V-0 grade was reached for the weight ratio 2:8 from the DGEBDB to the DGEBA system. Unfortunately, eugenol-based epoxy resin, due to its specific processing depending on the type of curing agent and complicated synthesis processes, requires intensive research on its structure [23]. Xue et al. synthesized EUEP epoxy monomer (EUEP) on the principles of the Diels–Alder addition reaction and epoxy–amine open-loop crosslinking. In the next step, ternary cure (EUEP-BDM-DDS) was performed with bismaleimide (BDM) and the curing agent 4,4′-diamino-diphenyl sulfone (DDS) [23]. This material exhibited a glass transition temperature T_g_ of 306 °C and superior mechanical properties with high moduli (up to 4.14 GPa for tensile and 4.10 GPa for flexural). The chemical structure and properties of the cured epoxy resin depend largely on the type of curing agent used. Moreover, the selection of the appropriate hardener depends on the processing conditions and the technology used, e.g., cycloaliphatic amines are less sensitive to moisture during processing than aliphatic ones. The novelty of this work lies in the analysis of the thermal properties of eugenol-based epoxy resins cured with aliphatic, aromatic, and cycloaliphatic amines. To date, there is little research available on the function of phosphorus associated with eugenol-derived biophenols in improving the thermal properties of epoxy resins cured with different types of amines. This allows us to state that precise thermal characterization of eugenol-based epoxy systems modified with phosphorus compounds and cured with three types of amines of different functionality is a new approach to the development of this field of science.

## 2. Results

### 2.1. Scanning Electron Microscopy (SEM)

**Figure 1:**
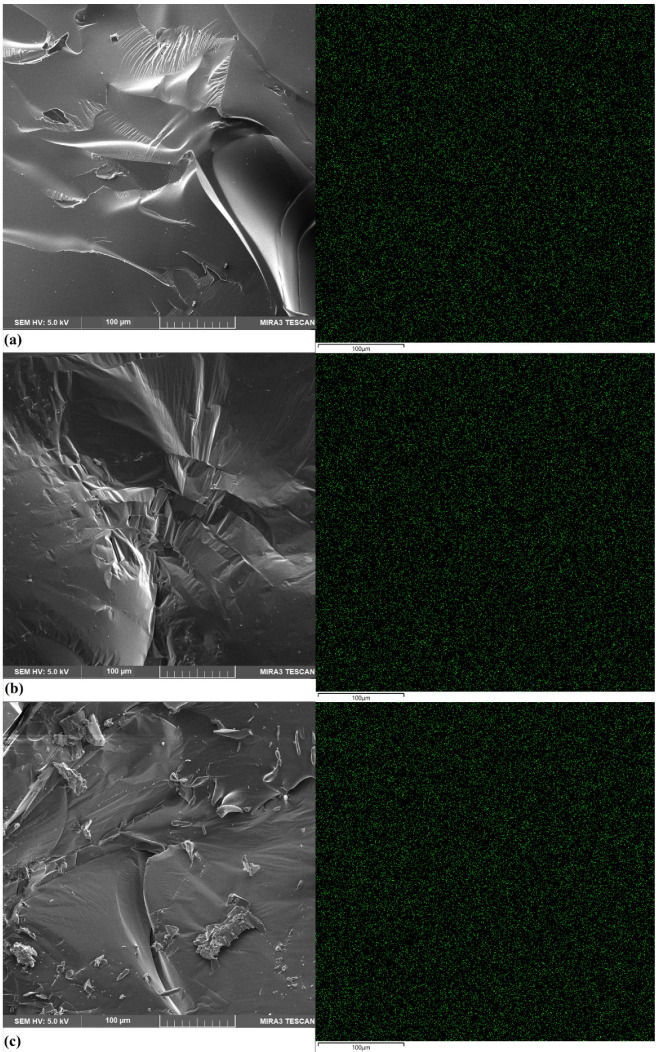
SEM photographs and P atom (green points) distribution map of (**a**) TEEP+TETA, (**b**) TEEP+DDM, and (**c**) TEEP+IDA.

**Figure 2:**
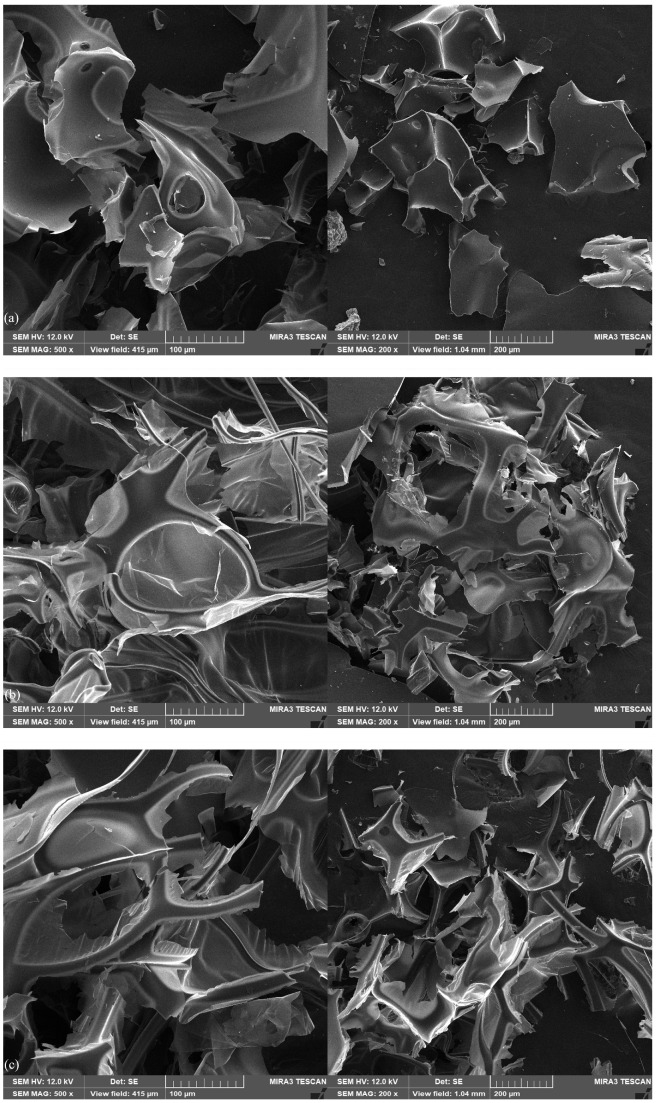
SEM photographs of char residue of (**a**) TEEP+TETA, (**b**) TEEP+DDM, and (**c**) TEEP+IDA.

The morphology of the cured bioresins is presented in Figure 1. The photos show brittle cracking of the epoxy structure and a map of the distribution of phosphorus atoms. A uniform dispersion of phosphorus atoms is visible on the sample surface, which is of great importance for the expected flame-retardant properties of this component. Modification of the epoxy resin structure can significantly increase the thermal stability, promoting the formation of a charcoal layer with a more cross-linked phosphorus structure and a smaller microcrystalline size. The surface morphology of the resulting ash after firing in a ceramic crucible in a nitrogen atmosphere was also assessed by SEM. In Figure 2, it can be observed that the surface of the tested bioresin is characterized by a spongy structure with some observable gullies, the formation of which may be the result of gas flow. The gases accumulated during degradation break the surface of the material and make it impossible to form a protective barrier in the form of char residue.

On this basis, it can be concluded that for the bioresins tested, a carbon layer is formed, which can slow the release of gases and reduce the thermal conductivity of the heat flow from the fire to the internal matrix. This phenomenon is also confirmed by the low heat release capacity (HRC) values recorded for the bioresin samples compared to the commercial epoxy samples. The carbon layer in this form more effectively insulates the substrate from thermal radiation and prevents the penetration of flammable gases into the fire, leading to reduced flammability [24,25]. Phosphorus compounds can combine with various types of aromatic compounds to promote thermal stability and the formation of a phosphorus-rich carbon layer [25,26].

### 2.2. TGA with FTIR

**Figure 3:**
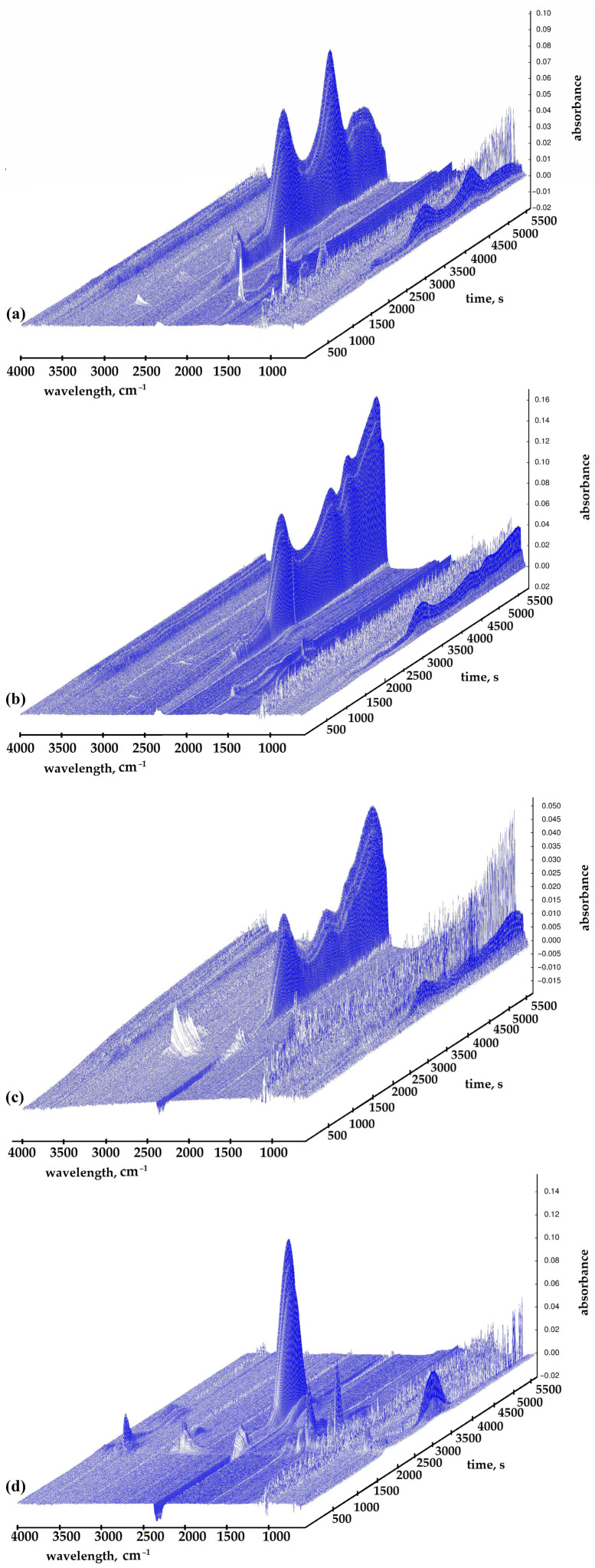
FTIR diagrams showing the evolution of gases as a function of time (blue) recorded during TGA for samples: (**a**) TEEP+TETA, (**b**) TEEP+DDM, (**c**) TEEP+IDA, and (**d**) DGEBA+TETA.

**Figure 4:**
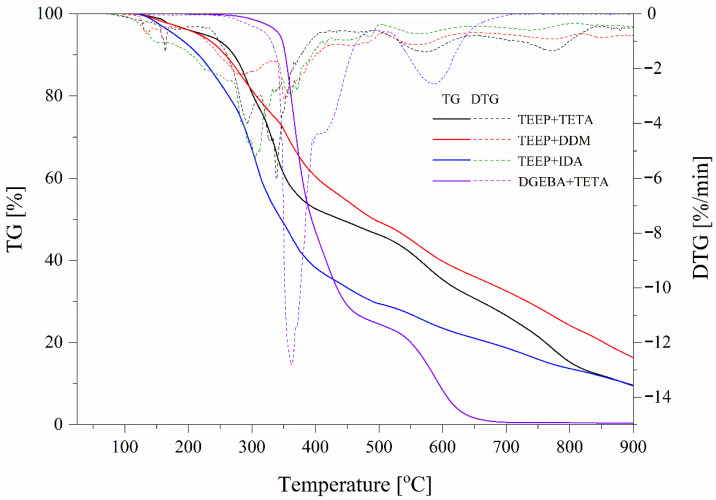
TGA and DTG curves of the tested samples.

During the TGA with FTIR, the purge gas is used to pass the volatile or decomposing products generated during the degradation of the material through metal pipes and glass [27]. The gas stream is then introduced into a spectrophotometer, where the components of the gas are detected. FTIR spectra of volatiles released during the decomposition of bioresins in the range of 100 to 995 °C in a nitrogen atmosphere are shown in Figure 3. The main groups observable on the curves are 2900, 2200–2400, 1800–1500, and 800–650 cm^−1^. The 2900 cm^−1^ is assigned to aliphatic C-H stretching vibrations. The bands corresponding to volatile components, such as CO_2_ (2360, 2344, 2310, and 670 cm^−1^), are observed. The region 2250–2400 cm^−1^ corresponds to the characteristic absorption band for the asymmetric stretching vibration of carbon dioxide (CO_2_). The region 2100–2200 cm^−1^ is designated to the stretching vibration of CO (carbon monoxide). In the 1650–1750 cm^−1^ range, the characteristic stretching vibration peak for the carbonyl group (C=O) is observed, and at 1510 cm^−1^, the characteristic stretching vibration peak for aromatic components is observed. It can be seen that after about 3500 s of the test, with increasing temperature, the peak intensity of the investigated materials at a wavelength of approximately 2400 cm^−1^ starts to grow, which is attributed to the stretching vibration of CO_2_ (2250–2400) and the stretching vibration of CO (2100–2200 cm^−1^) [28]. Compared to commercial resin (DGEBA+TETA), it can be seen that all tested bioresin samples release different nonflammable gases, which can protect the material during a fire. In addition, Biswas et al. proved that the introduction of phosphorus-based flame retardants decreases the resin degradation and enhances char formation at higher temperatures [29]. Epoxy resin has low thermal conductivity due to phonon scattering and the disordered orientation of its molecular chain [30]. The presence of flame retardants, acting primarily in the gas phase, in the resin matrix or structure can be effective in modifying the flame retardant. The flame-retardant additive can terminate reactive radicals to complete combustion reactions and thus balance flame retardancy and thermal conductivity [30]. The results of the TGA/FTIR analysis for TEEP samples and commercial resin are summarized in Table 1 and presented in Figure 4.

The residual mass during TGA is significantly higher and is 9.47% for TEEP+TETA, 16.33% for TEEP+DDM, and 9.61% for TEEP+IDA compared to the DGEBA+TETA sample, for which it is only 0.41%. Furthermore, the maximum degradation rate determined from the DTG curves for bioresin is significantly lower and is 6.1%/min for TEEP+TETA, 3.1%/min for TEEP+DDM, and 5.3%/min for TEEP+IDA compared to the DGEBA+TETA sample, for which it is 12.8%/min. These results may confirm that the presence of a phosphorus atom in the structure of the tested bioresin promotes the degradation of epoxy resin in advance and the formation of char at high temperature [30]. To achieve the most effective effect, flame retardants must be adapted to the type of thermal degradation that occurs in the polymer [30]. The layer of char formed during degradation on the surface of the epoxy resin can act as a protective shield over the material, which protects it from subsequent burning [31]. The improvement in charring of the TEEP-based material vs. DGEBA-based resin during its thermal decomposition may be due to the higher linking rate of the aromatic rings, which favors the charring. Also, for this reason, DDM-hardened compositions are characterized by higher amounts of char residue [32]. These results indicate the release of nonflammable gases, which can be combined with the results of the analysis of gases released by TGA-FTIR. The increased release of gases, such as those observed in the gas phase FTIR measurements, highlights the flame dilution potential of these materials [33]. In turn, TEEP-based epoxy resins exhibit lower thermal stability than commercial epoxy resins due to the lower binding energy of the –P–O bond compared to the –C–C bond [34].

The limiting oxygen index (LOI) value is an indicator to estimate the flame-retardant property of resins [35]. The LOI value of bioresin can be calculated according to the Van Krevelen and Hoftyzer equation [36]:

LOI = 17.5 + 0.4∙R_800_ (1)

where R is the weight residue at 800 °C of the cured epoxy samples.

The weight residue for samples recorded at 800 °C amounts to 15.29% for TEEP+TETA, 24.19% for TEEP+DDM, 13.68% for TEEP+IDA, and 0.48% for DGEBA+TETA, resulting in the following LOI values: 23.62% for TEEP+TETA, 27.18% for TEEP+DDM, 22.97% for TEEP+IDA, and 17.69% for DGEBA+TETA. Materials with an LOI value higher than 21% may be considered flame-retardant [35,37]. All the bioresin samples may be described as flame-retardant materials.

### 2.3. Pyrolysis and Combustion Flow Calorimeter (PCFC)

**Figure 5:**
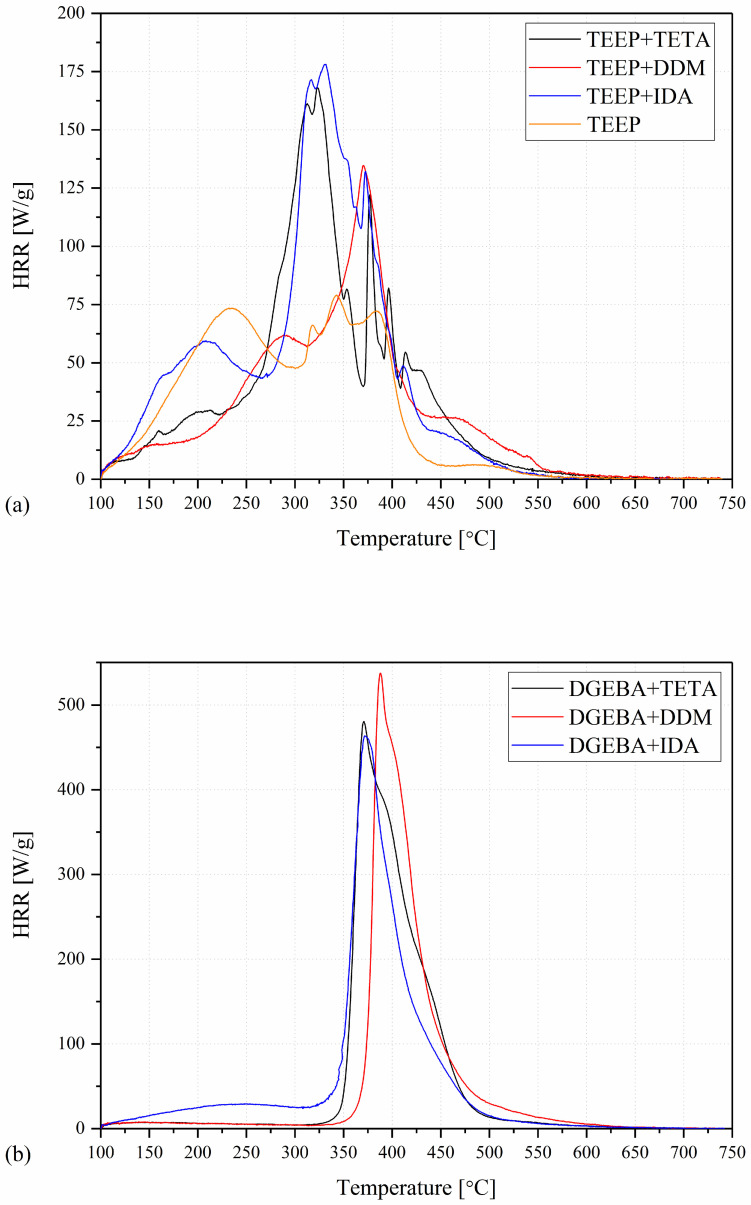
Heat release rate (HRR) as a function of temperature: (**a**) TEEP+TETA, TEEP+DDM, TEEP+IDA, and TEEP; (**b**) DGEBA+TETA, DGEBA+DDM, and DGEBA+IDA.

Comparison of the results of the PCFC analysis with those from thermogravimetric analysis allows for a better understanding of the rules of thermal degradation of polymeric materials. The bioresin and commercial epoxy resin samples cured with different amines were investigated. The samples are characterized by a different number of decomposition stages, as shown in Figure 5. The materials tested on commercial resin (DGEBA) are mostly characterized by a single-stage decomposition. Only for the DGEBA+IDA material can an additional stage be observed at the beginning of combustion. In turn, for bioresin samples, 5 to 7 peaks are observed in the microcalorimetric curves. This indicates a more complex phenomenon that occurs during the thermal degradation of these materials. A larger number of peaks on the HRR curves may indicate a typical intumescent effect during the combustion [38]. The results obtained are summarized in Table 2. One of the most important parameters that allows the evaluation of the reaction to material fire is the heat release rate (HRR), which is the most important factor in controlling fire hazards [39]. The heat release rate may be described as the mass loss rate of the material multiplied by its heat of combustion. After the peak heat release rate is reached, the HRR gradually decreases with time as a result of the formation and thickening of a surface char layer, which slows the rate of the decomposition reaction in the underlying material. It can be observed that for the bioresin samples, both p_c_HRR and T_pc_HRR values are much lower than for the DGEBA-based resin. It can also be observed that the type of curing agent used also influences the values discussed. The lowest p_c_HRR values are recorded for the liquid monomer TEEP.

The TEEP monomer is followed by the TEEP+DDM, TEEP+TETA, and TEEP+IDA samples, respectively, averaging 60 W/g, 132 W/g, 165 W/g, and 185 W/g. The highest values of p_c_HRR are recorded for the DGEBA+DDM sample. The increase in heat release rate may indicate a more complete pyrolysis and volatilization of the DGEBA resin matrix and a higher loss in mass as a result of its lower char yield. These results are in good agreement with those obtained in TGA. All bioresin samples are characterized by high char residue. The gas products that occur during the decomposition of organic components in polymer materials influence the amount of heat released [40]. Most polymers release CO and CO_2_ during the decomposition process [29]. The reduction in the heat release rate may also result from the reaction of the decomposition of organic components. Further effects, such as changes in the surface emissivity of the material during its decomposition, the specific heat of the reaction gases (which may have a convective cooling effect), and changes in the heat flux and oxygen content in the fire, have an impact on the heat release rate. The relationship between the HRR value and the rate of increase in sample temperature during the test is the basis for estimating the HRC value of the tested materials [41]. In turn, THR describes the amount of heat a polymer or material releases in a fire. For polymers with flame retardants, these values should be low [41,42]. This tendency is also observed for the bioresins tested. The lowest THR value is observed for the samples of TEEP (17.2 kJ/g) and TEEP+DDM (17.5 kJ/g).

## 3. Materials and Methods

### 3.1. Materials

Phosphorus-modified eugenol-based epoxy resin was synthesized from the following reagents: eugenol for synthesis (99%), phosphorus (V) oxychloride (99%), 3-chloroperbenzoic acid (≤77%; m-CPBA), triethylamine (≥99.5%; Et_3_N), ethyl acetate (≥99.5%; EtOAc), sodium bicarbonate (≥99.7%), anhydrous sodium sulfate (99%), and 4,4’-diaminodiphenylmethane (≥99.7%; DDM). All reagents were purchased from Sigma Aldrich (Poland). Other hardeners, such as triethylenetetramine (TETA) and isophorone diamine (IDA), were purchased from CIECH Sarzyna S.A., Podkarpackie, Poland. Commercial resin based on DGEBA (Epidian 5 produced by CIECH Sarzyna S.A., Podkarpackie, Poland) was used to prepare reference samples.

### 3.2. Synthetic Route to Obtain Tris(2-Methoxy-4-(2,3-Epoxypropyl)Phenyl Phosphate) (TEEP)

**Figure 6:**
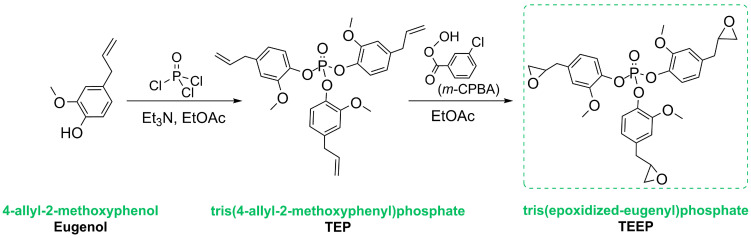
The reaction scheme for obtaining a bioepoxy resin monomer. i.e., tri(epoxized-eugenyl)phosphate (TEEP).

Tris(2-methoxy-4-(2,3-epoxypropyl)phenyl phosphate) (TEEP) was obtained by a synthetic protocol based on two reaction sequences presented in previous studies and according to Faye et al. [43,44]. This route involves a two-step reaction process, as outlined in Figure 6. The first step concerns the substitution of chlorine atoms in POCl_3_ with eugenol phenolic hydroxyl groups of eugenol, resulting in the formation of the phosphate ester (TEP). Subsequently, trieugenylphosphate (TEP) undergoes an epoxidation with *m*-chloroperoxybenzoic acid (m-CPBA), an oxidizing agent. The synthesized properties of TEEP, including its spectral and spectrometric data, align with the data reported in the literature, confirming the successful synthesis and purity of the compound [43,44]. The product was characterized by ^1^H, ^13^C, and ^31^P NMR, ESI MS, and elemental analysis techniques. ^1^H NMR (300 MHz, CDCl_3_): δ (ppm) 7.32 (3H, m Ph); 6.68–6.86 (6H, Ph); 3.76 (9H, OCH_3_); 3.12 (3H, epoxy CH–O); 2.81 (6H, CH_2_–CH–O); 2.78 (3H, epoxy CH_2_–O), 2.52 (3H, epoxy CH_2_–O). ^13^C NMR (75.48 MHz, CDCl_3_): δ (ppm) 151.40 (C_Ph_–OCH_3_); 138.13 (C_Ph_–O–P); 136.79 (C_Ph_–CH_2_); 121.00 (C_Ph_); 120.60 (C_Ph_); 113.67 (C_Ph_-OCH_3_); 55.99 (OCH_3_); 52.57 (C_epoxy_), 46.76 (C_epoxy-_CH_2_), 38.23 (CH_2_–CH–O). ^31^P NMR (162.02 MHz, CDCl_3_): −15.92 (O=PO_3_). High-resolution mass spectrometry (HRMS) (positive-ion electrospray ionization (ESI+)) *m*/*z* calculated for [C_30_H_33_O_10_P+H]^+^ 585.1890 found 585.1887. EA: Anal. calcd for C_30_H_33_O_10_P (%):C, 61.64; H, 5.69; found: C, 61.40; H, 5.60.

The reaction scheme is presented in Figure 6. The names of the compounds and the main product are highlighted in green.

### 3.3. Sample Preparation

Due to the specific processing and specified properties, different types of amine curing agents were used: aliphatic, aromatic, and cycloaliphatic. Therefore, in our research, bioresin was cured using three types of curing agents: triethylenetetramine (TETA), diaminodiphenylmethane (DDM), and isophorone diamine (IDA). The amount of curing agent (ma) was determined based on Equation (2), where AHEW is amine hydrogen equivalent weight, and EEW is epoxy equivalent weight.

(2) $$m_{a}=\frac{{AHEW}_{amine}}{{EEW}_{epoxy}}\cdot m_{epoxy}$$

The curing agent and the resin were stirred mechanically. The composition was cast in a Teflon mold and cured for 24 h at ambient temperature and then post-cured at 150 °C for 2 h. In the case of the DDM curing agent, which melts above a temperature of 90 °C, it was introduced into hot resin (100 °C) and mechanically mixed.

### 3.4. Characterization

***Nuclear magnetic resonance (NMR)***

NMR spectra were recorded on a Bruker Ascend™ 300 MHz operating at 300 MHz for ^1^H NMR, at 101 MHz for ^13^C NMR, and at 162 MHz for ^31^P NMR. Chemical shifts (*δ*) are reported in ppm. Reference values for residual solvent were taken as *δ* = 7.26 ppm (CDCl_3_) for ^1^H NMR and *δ* = 77.16 ppm (CDCl_3_) for ^13^C NMR.

***High-resolution mass spectrometry (HRMS)***

HRMS measurements were performed using a Synapt G2-Si mass spectrometer (Waters Corp., Milford, MA, USA) equipped with an ESI source and quadrupole-Ttime-of-flight mass analyzer. The mass spectrometer was operated in the negative ion detection mode.

***Elemental analysis***

Elemental analysis was performed using the Flash 2000 analyzer and based on dynamic combustion technology.

***Scanning electron microscopy (SEM)***

The microstructure of the samples was observed with an EDS-equipped scanning electron microscope (Mira 3, Tescan, the Czech Republic; Oxford Instruments Ultim Max 65, UK). Approximately 20 nm of carbon coating was deposited on samples using a JEE 4B vacuum evaporator (Jeol USA, USA) to reduce sample charging. The phosphorus concentrations are presented using color-scale EDS maps. The EDS results are presented as qualitative maps of the distribution of the concentrations of phosphorus.

***Thermogravimetric analysis (TGA) coupled to a Fourier transform infrared spectrometer (FTIR)***

Thermal stability analysis of cured samples was assessed using a thermal analyzer coupled to an FTIR spectrometer (STA 6000, Perkin Elmer, Waltham, Massachusetts, USA). The measurement was carried out in a nitrogen atmosphere at a heating rate of 10 °C/min, a gas flow rate of 20 mL/min, and a temperature range of 25–995 °C. From the curves obtained, the temperatures at which 5% and 10% mass loss were observed (T5% and T10%), and the residual mass (ΔW%) at 900 °C were determined. Furthermore, the maximum temperature and the thermal degradation rate were determined from the first derivative of the TGA curve (DTG curve).

***Pyrolysis and combustion flow calorimeter (PCFC)***

Flammability tests of bio-based resin were performed using a pyrolysis and combustion flow calorimeter (PCFC) by Fire Testing Technology Ltd. (East Grinstead, West Sussex, UK) according to ASTM D7309-2007. The decomposition was carried out in an inert gas atmosphere in the temperature range of 150–750 °C with a heating rate of 1 °C/s. The gases generated as a result of pyrolysis were then oxidized in a high-temperature furnace at 900 °C to completely oxidize. The following parameters were determined: heat release rate (HRR) in the form of a peak for the entire sample (pcHRR), temperature of the HRR peak for the entire sample (T_pc_HRR), total combustion heat (THR), and heat release capacity (HRC).

## 4. Conclusions

In conclusion, we have shown that eugenol can be successfully used to produce epoxy resin with enhanced thermal properties. As a result of combining eugenol with a phosphorus compound, a material was obtained that, during thermal degradation, is covered with a significant amount of char residue and is characterized by a reduced value of heat release rate (HRR) and heat release capacity (HRC) in comparison with the resin based on the petrochemical DGEBA. The results obtained provide valuable information on the degradation characteristics of eugenol-based bioresins and flame properties and their potential application at high temperatures. The research is part of ongoing work and may be the basis for further discoveries in the use of raw materials from sustainable sources for the synthesis of chemically cured resins.

## Figures and Tables

**Table 1:** Thermogravimetric data of the investigated samples in an inert atmosphere.

Name	T5% (°C)	T10% (°C)	Residual Mass (%)	DTG Peak (°C)/max. Rate (%/min)
TEEP+TETA	224.5	271.6	9.47	338.7/6.1
TEEP+DDM	216.8	258.2	16.33	350.7/3.1
TEEP+IDA	178.2	264.6	9.61	304.7/5.3
DGEBA+ TETA	343.4	352.6	0.41	336.1/12.8

**Table 2:** Microcalorimetric data of the tested materials.

Name	pcHRR (W/g)	TpcHRR (°C)	THR (kJ/g)	HRC (J/g∙K)
TEEP	59.7 ± 2.9	205 ± 8	17.2 ± 0.5	84 ± 3
TEEP+TETA	165.3 ± 7	317 ± 5	20.6 ± 0.7	182 ± 5
TEEP+DDM	132.2 ± 4.1	369 ± 2	17.5 ± 0.3	144 ± 4
TEEP+IDA	185 ± 20.0	320 ± 5	24.0 ± 0.7	203 ± 9
DGEBA+TETA	461.5 ± 34.1	373 ± 2	31.5 ± 1.4	501 ± 32
DGEBA+DDM	548.5 ± 22.5	391 ± 2	29.6 ± 0.2	597 ± 29
DGEBA+IDA	461.4 ± 24.4	373 ± 2	32.1 ± 1.3	518 ± 32

## Data Availability

The data supporting the findings of this study are available from the corresponding author (Danuta Matykiewicz) on request and in the following dataset: https://doi.org/10.18150/BGX9CO (accessed on 30 August 2025).
